# A Representative Clinical Course of Progression, with Molecular Insights, of Hormone Receptor-Positive, HER2-Negative Bone Metastatic Breast Cancer

**DOI:** 10.3390/ijms25063407

**Published:** 2024-03-17

**Authors:** Elizabeth Magno, Karen M. Bussard

**Affiliations:** Department of Pharmacology, Physiology and Cancer Biology, Thomas Jefferson University, Philadelphia, PA 19107, USA

**Keywords:** breast cancer, bone, bone metastasis, tumor microenvironment, chemotherapy, endocrine therapy, radiation therapy, bone modifying agents, bisphosphonates

## Abstract

Despite treatment advances, breast cancer remains a leading cause of death of women in the United States, mostly due to metastatic disease. Bone is a preferential site for breast cancer metastasis, and most metastatic breast cancer patients experience bone involvement at the time of death. The majority of patients with bone metastatic breast cancer are first diagnosed with and treated for early-stage disease, and from development of early-stage breast cancer to the recurrence of cancer in the bones, up to 30 years may elapse. Throughout this timeframe, a typical patient undergoes many treatments that have effects on the bone microenvironment. Therefore, this review explores the clinical course of a representative patient with hormone receptor-positive bone metastatic breast cancer, examining key treatment options at each stage and their effects on preventing and treating bone metastases.

## 1. Introduction

Breast cancer is the most frequently diagnosed malignancy among women in the United States, excluding skin cancers [1]. Approximately 1 out of every 8 (13%) women will be diagnosed with breast cancer during their lifetime [1]. Breast cancer is the second leading cause of death among women in the United States, and although the disease predominately affects women, 1% of breast cancer cases and deaths occur in men [1]. In 2024 alone, 42,780 patients are expected to die from the disease [2]. The majority of breast cancer-related deaths occur from metastatic disease, characterized by the spread of cancer cells from the breast to other tissues, leading to their proliferation at secondary sites [3]. Up to 30% of patients diagnosed with breast cancer will ultimately develop metastases [4]. Common metastatic sites include the bones, lungs, liver, and brain, with bone being the most prevalent location [5]. Among all patients who die from breast cancer, approximately 73% exhibit bone involvement at the time of death, representing the highest percentage of bone involvement among all cancers [6]. While each patient’s journey is unique, this review illustrates a representative case of bone metastatic breast cancer, featuring a patient with the most statistically common clinical features: Hormone Receptor (HR) positive, Human Epidermal growth factor Receptor 2 (HER2)-negative breast cancer without germline mutation. From development of early-stage breast cancer to the recurrence of cancer in the bones, up to 30 years may elapse [7]. A common clinical course of progression is presented, highlighting key events, including presentation to the clinic, primary and adjuvant treatment involving locoregional and/or systemic therapy, progression to metastasis, and treatment of bone metastatic breast cancer. At each juncture, therapeutic options and their effects on the bone microenvironment are discussed.

## 2. Presentation to Clinic, Imaging, Biopsy, and Staging

While a minority of patients initially present with widespread metastatic disease, 92% of breast cancers in the United States are diagnosed in local or regional stages [1]. Importantly, among patients who ultimately develop metastatic breast cancer, 74% are initially diagnosed with early-stage breast cancer [8]. Patients with early-stage, primary breast cancer often present to the clinic after discovering a palpable mass within the breast, either through self-examination, clinician detection, or a suspicious finding on screening mammography. Diagnostic mammography is then used to identify the location and relative size of the primary tumor. In individuals with dense breasts, breast ultrasound and Magnetic Resonance Imaging (MRI) may also be used to better visualize the suspected tumor [9]. A sample of the primary tumor is then obtained via core biopsy, fine needle aspiration, or incisional biopsy and profiled in order to assess tumor hormone-receptor status, molecular markers and mutational status, and histology [9]. These characteristics from biopsy tissue are synthesized to inform treatment decisions and determine a patient’s eligibility for certain therapies.

Tumor expression of hormone receptors is critical in determining both prognostic and therapy predictive information [10]. Cancers with as few as 1% of cells positive for Estrogen Receptor (ER) or Progesterone Receptor (PR) expression via ImmunoHistoChemistry (IHC) staining are considered ER or PR-positive, respectively [11]. Cancers that are either ER-positive, PR-positive, or both are considered Hormone Receptor (HR)-positive. ER itself is a DeoxyriboNucleic Acid (DNA)-binding transcription factor that is responsive to its ligands, including estrogen, the main hormone regulating mammary gland development. Estrogens are believed to stimulate breast cancer cell growth by associating with regulatory elements in the genome, enhancing the transcription of genes such as MYeloCytomatosis (*MYC*) and CyCliN D1 (*CCND1*) and promoting tumor cell growth and proliferation [12]. Because ER-positive breast cancers are responsive to circulating estrogens which stimulate breast cancer cell growth, suppression of endogenous estrogen and consequent abrogation of ER-signaling is a critical part of ER-positive breast cancer management. Meanwhile, PR is dually expressed in more than 50% of ER-positive cancers [13]. PR is an ER-induced gene, and PR positivity is thought to reflect a cancer that is driven by an active ERα complex and therefore likely to respond to endocrine therapies [14]. Patients with ER-negative, PR-positive cancers may therefore be considered for endocrine therapies, but data on this group are limited [9].

Along with HR status, HER2 expression is among the most important clinical and prognostic factors to determine for breast cancer patients. *HER2* is a membrane tyrosine kinase and oncogene that is overexpressed and gene amplified in about 20% of breast cancers [15]. Transcription factors activated by the HER2 pathway regulate genes involved in cell proliferation, survival, differentiation, angiogenesis, and invasion and metastasis [15]. *HER2* amplification and resultant overexpression confer clinical aggressiveness, predicting poorer overall survival and shorter time to relapse [16]. However, patients with tumors overexpressing HER2 protein are candidates for HER2-targeted therapies, including adjuvant trastuzumab, which significantly improves disease-free survival [9]. Therefore, determining HER2 expression via IHC is a critical component of molecular tumor diagnostics. HR-positive/HER2-negative breast cancers are by far the most common subtype in the United States, comprising 68% of all diagnosed invasive female breast cancers, followed by HR-positive/HER2-positive and HR-negative/HER2-negative (triple negative) breast cancer, each at ten percent overall [1]. HR-positive/HER2-negative cancers are significantly associated with bone relapse compared with HER2-positive or HR-negative subtypes [17]. In addressing bone metastatic breast cancer, HR-positive/HER2-negative patients are therefore among the most vulnerable populations to focus on.

The genomic landscape of breast cancers is heterogeneous but follows a general pattern, with tumors broadly classified into four intrinsic subtypes: luminal A, luminal B, HER2-enriched, and basal-like (also termed triple-negative) [18]. Each subtype bears specific natural gene expression patterns by gene expression array, and subtypes differ in natural histories, response to therapy, and epidemiologic frequencies [19]. 

Furthermore, some breast cancer subtypes are associated with particular somatic mutations; somatic mutations in only three genes (Tumor Protein P53 (*TP53*), Phosphatidylinositol-4,5-Bisphosphonate 3-Kinase Catalytic Subunit Alpha (*PIK3CA*), and GATA Binding Protein 3 (*GATA3*)) are represented at >10% incidence across all subtypes [20]. Luminal breast cancers have the most heterogeneous mutations, with Luminal A cancers demonstrating *PIK3CA* mutations most frequently, followed by Mitogen-Activated Protein Kinase Kinase Kinase 1 (*MAP3K1*), *GATA3*, *TP53*, Cadherin 1 (*CDH1*), and Mitogen-Activated Protein Kinase Kinase 4 (*MAP2K4*) alterations, while luminal B cancers have frequent *TP53* and *PIK3CA* mutations (29% each). In contrast, basal-like cancers have few shared mutations with the luminal subtypes, and instead are characterized by a very high frequency of *TP53* mutations (84%) and loss of function [20].

Microarray analysis to determine the intrinsic subtype of a given tumor is costly, difficult to standardize, and not commercially available in the United States, so intrinsic subtype classification is limited to research settings [21]. Clinically, expression of ER, PR, and HER2 by IHC staining is often used as a surrogate for intrinsic subtype classification, with luminal subtypes defined by presence of ER staining (highest in A and lower in B), HER2-enriched by HER2 positivity, and basal-like by absence of ER, PR, and HER2. However, these clinical markers are a poor heuristic for intrinsic subtype determination. For example, when analyzed by microarray, 5% of clinical Luminal A samples were actually the basal-like subtype, and 30% of HER2-negative tumors were the HER2-enriched subtype [22]. Therefore, clinicians use factors other than intrinsic subtype to stratify risk, including multigene assays such as OncoType Dx, MammaPrint, Prosigna, EndoPredict, and Breast Cancer Index (BCI) to assess genomic level information that predicts recurrence risk and/or potential therapy benefit for certain populations [23]. 

Beyond tumor-level genomic analysis, germline genetic testing may also be performed in a newly diagnosed patient to evaluate hereditary breast cancer genes [9]. In contrast to somatic mutations, which develop in specific cells over time, germline mutations are inherited and are present in every cell throughout the body. Often, these germline mutations disrupt the normal functioning of processes like DNA repair or regulation of cell proliferation, thereby predisposing patients to cancer development [24]. Most significantly, inherited mutations in the DNA repair genes BRCA1/2 DNA Repair Associated (*BRCA1/2*) incur a lifetime risk of breast cancer of approximately 50% [24]. Pathogenic germline variants in Partner And Localizer of BRCA2 (*PALB2*), ATM Serine/Threonine Kinase (*ATM*), BRCA1 Associated RING Domain 1 (*BARD1*), and Checkpoint Kinase 2 (*CHEK2*) individually increase breast cancer risk, while *TP53*, Phosphatase And Tensin Homolog (*PTEN*), Serine/Threonine Kinase 11 (*STK11*), Neurofibromin 1 (*NF1*), and *CDH1* mutations increase breast cancer risk in association with genetic syndromes [24,25]. Some germline mutations make patients eligible for targeted therapies, such as poly adenosine diphosphate (ADP)-ribose polymerase (PARP) inhibitors in patients with *BRCA1/2* mutations. Although germline mutations are more common within certain ethnic groups, they have low prevalence among the general population (<7% for BRCA1 DNA Repair Associated (*BRCA1*), the most common germline mutation) [26].

At diagnosis, in addition to surveying the molecular characteristics of the primary tumor, a patient with breast cancer is also assessed for cancer spread. Axillary lymph nodes (ALNs), which drain lymph fluid from the breast, are biopsied in order to determine if cancer cells have spread from the primary tumor into secondary locations via the lymphatic system. If signs or symptoms of metastatic disease are present, systemic imaging is used to evaluate the extent of metastasis. The patient’s cancer is then staged using the traditional anatomic Tumor (T), Lymph Node (N), Metastases (M) (TNM) system, based on the size of the primary tumor, the number of regional lymph nodes with cancer cells present, and the number of distant metastases. Tumors are also graded based on histological stage, which is determined based on tissue architectural distortion in biopsy samples. The TNM staging, histological staging, molecular markers, and HR status are synthesized to generate both clinical and pathological prognostic stages.

## 3. Treatment of Primary Hormone Receptor-Positive Breast Cancer

**Figure 1:**
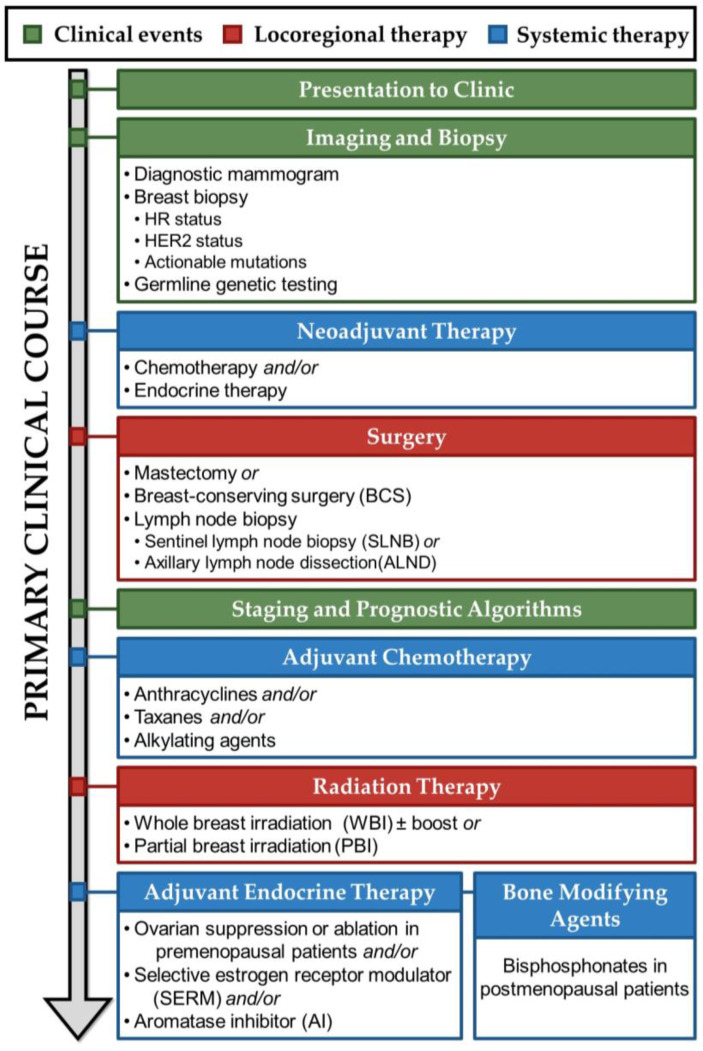
Representative timeline of primary breast cancer clinical course for a hormone receptor-positive, HER2-negative patient. Following presentation to the clinic, a patient undergoes diagnostic imaging and breast biopsy, on which molecular testing is performed. Neoadjuvant therapy may be employed to reduce tumor burden. Next, surgery removes the primary tumor, along with all or part of the breast, and lymph nodes are surveyed for spread of cancer cells outside of the breast. Information garnered from the breast and lymph node biopsies are synthesized using staging and prognostic algorithms, which help health care providers decide which therapeutics to administer. Adjuvant chemotherapy may be administered to reduce the risk of later cancer recurrence, followed by radiation therapy of the local site. Finally, adjuvant endocrine therapy is administered concurrently with bone modifying agents to reduce cancer recurrence risk and deleterious effects on skeletal health.

The therapeutic management of primary breast cancer is complex, varied, and multimodal. Treatment may begin with neoadjuvant systemic therapy but almost always includes local therapy of the affected breast, such as surgery in order to remove the primary tumor, and subsequent radiation therapy (RT) to eradicate any remaining cancer cells in the breast. Whole-body systemic therapy, such as chemotherapy, endocrine therapy, targeted therapy, or immunotherapy, is then often used to kill any remaining cancer cells and reduce the risk of later recurrence. For such cases, primary cancer treatment involves a sequential approach: neoadjuvant hormone therapy to shrink a tumor, then surgery, followed by adjuvant systemic therapy and radiation, then several years of adjuvant endocrine therapy (Figure 1) [9].

### 3.1. Neoadjuvant Hormone Therapy for Primary Hormone Receptor-Positive Breast Cancer

Prior to surgery, neoadjuvant therapy may be used for patients with hormone receptor-positive breast cancer, in order to reduce the size of a patient’s primary tumor for future surgical removal [27]. Both chemotherapy and endocrine therapy may be employed in the neoadjuvant setting. In particular, endocrine therapy reduces the amount of circulating estrogen and/or progesterone, and can only be used for patients with hormone receptor-positive cancers. This is because HR-positive cancers are responsive to estrogen and progesterone [28]. Most often, neoadjuvant endocrine therapy is used in post-menopausal women, and it is especially advantageous for patients with larger tumors [29]. By reducing the size of the tumor, surgeries that spare more breast tissue, such as a lumpectomy, may be used instead of mastectomy [27,30]. Furthermore, neoadjuvant endocrine therapy may also provide prognostic information based on a patient’s response, which can better inform adjuvant treatment regimens [23,31]. 

Common neoadjuvant endocrine therapies include either tamoxifen or aromatase inhibitors, such as letrozole, anastrozole, or exemestane. Importantly, comprehensive studies have been carried out to examine the efficacy of aromatase inhibitors versus tamoxifen as neoadjuvant treatments. In both the Immediate Preoperative Anastrozole, tamoxifen, or Combined with Tamoxifen (IMPACT) and PRe-Operative “Arimidex” Compared to Tamoxifen (PROACT) trial, the aromatase inhibitor anastrozole was as effective, and in some cases more effective than tamoxifen for reducing tumor volume [29,32]. Furthermore, letrozole has been shown to be more effective than tamoxifen as a neoadjuvant treatment in post-menopausal women with primary hormone receptor-positive breast cancers [33]. Thus, neoadjuvant endocrine therapy that uses an aromatase inhibitor may offer more benefit for patients than tamoxifen.

In some patients with hormone receptor-positive, HER2-negative breast cancer, chemotherapy may also be used as a neoadjuvant treatment [34]. There is one main consideration when deciding if chemotherapy should be used as a neoadjuvant treatment (as opposed to post-surgical treatment): is it necessary to obtain pathological or genomic information from a patient’s tumor to help select an appropriate chemotherapeutic agent? If no pathological or genomic information is needed, neoadjuvant chemotherapy is appropriate to use [34]. If genomic and/or pathological information about the tumor is needed, however, then chemotherapy should be used after surgery [34]. Interestingly, a landmark clinical trial showed no difference between overall survival for the use of certain neoadjuvant or adjuvant chemotherapy regimens [35]. 

### 3.2. Locoregional Therapy for Primary Hormone Receptor-Positive Breast Cancer

Patients and clinicians have a number of surgical options for removal of breast tumors. Breast Conserving Surgery (BCS), commonly referred to as lumpectomy, entails the removal of the visible tumor and some surrounding normal-looking tissue, while preserving the remaining breast tissue. After tumor removal, surgical margins are microscopically evaluated, and if cancer cells are detected, a second surgery removes additional breast tissue around the tumor site. BCS is followed by radiation therapy of the affected breast, with or without regional lymph nodes, to eradicate any remaining malignant cells. The alternative to BCS is mastectomy, which removes all of the breast. Total mastectomy, also termed simple mastectomy, removes the entire breast, while radical mastectomy removes all breast tissue, axillary lymph nodes along the breast, and, when necessary, underlying chest wall muscle. Mastectomy is indicated in cases of widespread tumors, diffusely positive margins, cancer spreading to chest muscles, and during pregnancy [9]. Selecting the appropriate surgical intervention entails shared decision-making between patients and clinicians, considering factors like long-term survival, the risk of local recurrence, and the impact on cosmetic outcomes and overall quality of life. Overall, BCS with radiation and mastectomy show similar overall survival outcomes for patients [36], though some studies suggest improved survival [37,38] and fewer post-surgical complications [39] with BCS. 

In addition to removing the primary tumor from the breast, nearby axillary lymph nodes are assessed to determine the extent of breast cancer cell spread. Axillary Lymph Node Dissection (ALND) is a traditional approach that removes and assesses at least 10 lymph nodes for the presence of breast cancer cells. However, ALND is associated with high complication rates, including lymphedema and sensory loss, and has largely been replaced by Sentinel Lymph Node Biopsy (SLNB) [40]. A Sentinel Lymph Node (SLN) is any lymph node that receives direct lymphatic drainage from a primary tumor site [40]. In SLNB, a radiopharmaceutical tracer and/or visible dye are injected peritumorially and monitored for spread to identify draining sentinel lymph nodes, which take up the tracer and dye. The SLNs are then excised and microscopically evaluated by Hematoxylin and Eosin (H&E) staining [41]. If the SLNs do not contain malignant cells, it is presumed that the cancer is contained within the breast. If cancer cells are identified, further ALND is indicated. Importantly, axillary lymph node status, not tumor size, predicts locoregional recurrence and overall survival [42]. Involvement of axillary lymph nodes at diagnosis is also the single greatest clinical risk factor for later development of bone metastasis [43]. Patients with axillary lymph nodes positive for cancer cells are therefore considered for systemic adjuvant treatment, Radiation Therapy (RT), and bisphosphonate therapy [9].

### 3.3. Systemic Therapy for Primary Hormone Receptor-Positive Breast Cancer

In contrast to locoregional treatments, which remove cancer within the breast and surrounding structures, systemic therapy treats the entire body to reduce the risk of cancer recurrence. The decision to administer adjuvant systemic therapy after primary treatments such as surgery is guided by the patient’s individual risk of relapse and the anticipated effectiveness of targeted treatments. Clinicians use algorithms incorporating prognostic factors like age, comorbidity, tumor size, grade, and the number of involved lymph nodes to estimate recurrence risk [31]. Additionally, multigene assays like Oncotype DX assess somatic mutations present within tumor tissue to determine the potential benefits of adding adjuvant chemotherapy to endocrine therapy for specific patient populations [23]. The potential survival benefits of adjuvant therapy are carefully weighed against considerations of toxicity and the patient’s existing comorbidities, in order to assess whether chemotherapy should be administered [9].

### 3.4. Adjuvant Chemotherapy for Primary Hormone Receptor-Positive Breast Cancer

Numerous cytotoxic chemotherapy regimens for breast cancer have undergone extensive evaluation in large-scale clinical trials to assess both efficacy and toxicity. Consequently, several preferred regimens have emerged for the treatment of early breast cancer [9]. While individual drugs within these classes may be interchangeable, three primary drug classes are commonly employed: anthracyclines, taxanes, and alkylating agents. 

Anthracyclines, exemplified by doxorubicin and its derivative epirubicin, integrate into the DNA of cancer cells, disrupting topoisomerase-II-mediated DNA repair. Additionally, they generate free radicals that inflict damage on cellular membranes, DNA, and proteins [44]. In preclinical mouse models, doxorubicin treatment causes bone loss through an interplay between oxidative stress and induction of Tumor Growth Factor-Beta (TGFβ) signaling [45]. TGF-β has both tumor-suppressive and tumor-promoting functions, but in the context of the cancer-bone microenvironment, it stimulates breast cancer cells to secrete ParaThyroid Hormone-Related Protein (PTHrP), which begins a “vicious cycle” that results in bone-resorbing osteoclast formation and activation [46]. This osteoclast-mediated bone destruction liberates growth factors stored in the bone matrix, fueling the proliferation of cancer cells [47]. Furthermore, patients treated with cyclophosphamide and doxorubicin undergo a reduction in Bone-Mineral Density (BMD) [48] with increasing bone resorption markers, which are predictors of bone metastasis [49]. It has also been demonstrated that primary breast cancer patients with low BMD have greater numbers of disseminated tumor cells in the bone than patients with normal bone density [50]. Therefore, the anthracycline-induced reduction in BMD that occurs during treatment of the primary breast tumor may contribute to later bone metastases by increasing bone turnover and allowing disseminated tumor cells to seed the bone.

Taxanes, such as docetaxel and paclitaxel, impede movement and function of microtubules, thereby altering cell migration and cell cycle progression [51]. The specific role of taxanes in bone homeostasis is not well studied [52]. 

Alkylating agents, including cyclophosphamide and platinum-based chemotherapies like carboplatin, create permanent cross-linkages within and between adjacent DNA strands, inducing apoptosis in cancer cells [53]. An adverse side effect of alkylating agents is inadvertent induction of menopause, which is defined as the permanent cessation of menses [54]. Premenopausal patients treated with modern regimens of alkylating agents are at intermediate risk of experiencing permanent chemotherapy-induced amenorrhea [55]. Cyclophosphamide, in particular, is highly gonadotoxic, causing a significant loss of ovarian primordial follicle reserve [56]. Anthracylines, too, pose an intermediate to high risk of permanent amenorrhea, with doxorubicin inducing DNA double-stranded breaks that cause primordial follicle apoptosis [55,57]. However, for both agents, patient age at the time of treatment, baseline ovarian reserve, and specific treatment regimen determine individual risk of ovarian toxicity [55]. 

Menopause, both natural and induced, leads to a precipitous drop in hormones levels as ovaries cease to synthesize estrogen and progesterone [58]. Estrogen is one of the main hormonal regulators of bone and causes pleotropic effects. Long-term estrogen deficiency in post-menopausal women leads to a slight increase in bone formation and a large increase in bone resorption, causing an overall net loss of bone mass [59]. Consequently, patients who experience chemotherapy-induced ovarian failure develop rapid and highly significant decreases in BMD that are detectable within 6 months of starting chemotherapy [58]. Decreasing BMD following the loss of estrogen is associated with increased expression of bone turnover markers [60], which are predictors for metastatic recurrence [49].

Preferred treatment regimens of cytotoxic chemotherapies in early breast cancer include (1) doxorubicin plus cyclophosphamide followed by paclitaxel (termed “AC-T”, after doxorubicin’s brand name, Adriamycin) and (2) docetaxel plus cyclophosphamide (termed “TC”, after one of docetaxel’s brand names, Taxotere). Trials examining outcomes of the two regimens have found them equivocal. However, no trials have examined the influence of each regimen on bone health and subsequent development of bone metastases [61].

### 3.5. Radiation Therapy for Primary Hormone Receptor-Positive Breast Cancer

After adjuvant chemotherapy, radiation may be employed. Whole Breast Irradiation (WBI) has been shown to reduce the risk of local recurrence and risk of death from breast cancer [62]. For patients with high-risk characteristics (such as age less than 50 years, high-grade disease, or focally positive margins), radiation to the tumor bed has been shown to reduce local relapse [63]. Radiation can also be applied to the chest wall and regional lymph node basins when indicated. In contrast to WBI, Partial Breast Irradiation (PBI), a targeted radiation therapy administered exclusively to the breast tissue surrounding the site of tumor removal, may be considered for certain low-risk patients [64]. Whether utilizing WBI or PBI, radiotherapy is highly localized to the area at risk of disease recurrence, and concerted effort is made to spare surrounding tissue and structures [9]. As such, nearby skeletal structures are rarely affected, though rib fracture can occur as a very rare, late complication [65].

### 3.6. Endocrine Therapy for Primary Hormone Receptor-Positive Breast Cancer

Following chemotherapy, endocrine therapy modulates the production or activity of a patient’s hormones to prevent HR-positive cancers from responding to them. Because HR-positive cancers are responsive to estrogen or progesterone, endocrine therapy seeks to reduce the amount of, mainly, circulating estrogen (as PR activity is driven partly, but not exclusively, by ER-mediated transcription, and the role of progesterone alone in breast carcinogenesis is not well defined) [28]. 

Tamoxifen is the most established adjuvant endocrine therapy. For those diagnosed with ER-positive breast cancer, the use of adjuvant tamoxifen leads to a 39% reduction in the annual likelihood of recurrence and a 31% decrease in the annual likelihood of mortality [66]. Importantly, these benefits remain consistent, regardless of factors such as chemotherapy utilization, patient age, menopausal status, or ALN status [66]. Tamoxifen belongs to a class of drugs called Selective-Estrogen Receptor Modulators (SERMs) that have mixed estrogen agonist or antagonist effects based on target tissue. Tamoxifen and raloxifene, another SERM member, both have estrogen antagonist effects in the breast and agonist effects in the bone, making them useful for both breast cancer and fracture prevention [67]. 

In patients who are premenopausal at diagnosis, endocrine therapies are often combined with ovarian suppression. The ovaries are premenopausal patients’ main source of estradiol, so abrogating ovarian function decreases estrogen production [68]. Therapeutic options include ovarian suppression or ovarian ablation [12]. Ovarian suppression utilizes pharmacotherapy to temporarily halt ovarian estrogen production. The most common agents, Luteinizing Hormone-Releasing Hormone (LHRH) agonists, suppress hormonal release from the pituitary gland and suppress estrogen production throughout the duration of drug administration [69]. After discontinuation, estrogen production and menstruation resume. Ovarian suppression is generally the preferred modality among premenopausal patients [69], but permanent ovarian ablation through either surgical oophorectomy or ovarian irradiation remains an important therapeutic option. Ovarian ablation may be employed to manage suboptimal ovarian suppression or instances of “breakthrough” ovarian function associated with pharmacologic agents. Additionally, it may be considered for patients with an inherited cancer syndrome elevating the risk of ovarian cancer or for those who do not wish to pursue pregnancy [70]. Importantly, among pre-menopausal women under the age of 45, both ovarian ablation and suppression significantly decrease the risk of recurrence and mortality from breast cancer, with no demonstrated distinction between the two methods [71].

Following therapy-induced ovarian suppression or after natural menopause, patients exclusively generate estrogen in extragonadal sites such as adipose tissue, kidneys, skin, and the brain [72]. While extragonadal estrogen generally functions locally as a paracrine factor, it can still contribute to the growth of ER-positive breast cancers [72]. Estrogen is a steroid hormone derived from androgen precursors, and the enzyme aromatase converts androstenedione and testosterone into estrone and estradiol [68]. Therefore, Aromatase Inhibitors (AIs) are used to block aromatase activity, impeding the production of estrogens and the growth of ER-positive breast cancers. AIs include anastrozole, letrozole, and exemestane. 

For HR-positive cancers, endocrine therapy is indicated in the majority of cases, regardless of menopausal status, age, or HER2 status of the tumor [12]. However, the specifics of endocrine treatment depend on these individual factors. Overall recommendations for adjuvant endocrine therapy are complicated, but generally consist of SERMs and/or AIs for up to 10 years [9]. 

## 4. Bone Remodeling and Its Dysregulation in Cancer

Bone is constantly being built and degraded in response to the body’s needs, including in response to mechanical stress and to maintain mineral homeostasis [73]. Bone remodeling is a tightly regulated process, mainly involving three different cell types: osteoblasts, which deposit new bone matrix; osteocytes, which are mature bone cells; and osteoclasts, which resorb bone [74].

Osteoblasts are the body’s bone-building cells and are derived from mesenchymal stromal cells located in the bone marrow [74]. Osteoblasts undergo a distinct differentiation process characterized by three main stages of growth: proliferation, extracellular matrix maturation, and extracellular matrix mineralization [75]. Each stage is marked by specific factor expression [75]. Once osteoblasts are fully differentiated, they are capable of laying down bone matrix, which is composed of hydroxyapatite, type I collagen, non-collagenous proteins, and water [74,76,77,78]. After synthesizing new bone matrix, an osteoblast either undergoes apoptosis or becomes embedded in the bone as an osteocyte [79]. Osteocytes are the mechanosensors of bone and communicate between osteoblasts and osteoclasts to initiate bone remodeling [80].

Osteoclasts are the bone cells responsible for matrix resorption. Osteoclasts are derived from the monocytes that are located in the bone marrow stroma [81]. Bone marrow monocytes are activated to form osteoclasts through ligands expressed on osteoblasts. Osteoblasts express the Receptor-Activator for NFκB Ligand (RANKL) on their membrane surface, as well as secrete soluble RANKL (sRANKL) [82]. Both sRANKL and RANKL can bind to the receptor RANK found on the surface of bone marrow monocytes. Osteoblasts also produce OsteoProteGerin (OPG), which is a decoy receptor for sRANKL and RANKL. Osteoclast formation is regulated by the ratio of OPG:RANKL [83]. Osteoblasts additionally produce Macrophage Colony Stimulating Factor (M-CSF), which, in the presence of RANKL, promotes cellular fusion of several monocytes to form one large, multinucleated osteoclast [81]. Activated osteoclasts then bind to the bone matrix through α_v_β_3,_ α_v_β_5_, α_2_β_1_ integrins located on the membrane surface and secrete acid and lysosomal enzymes to degrade bone [79,81].

**Figure 2:**
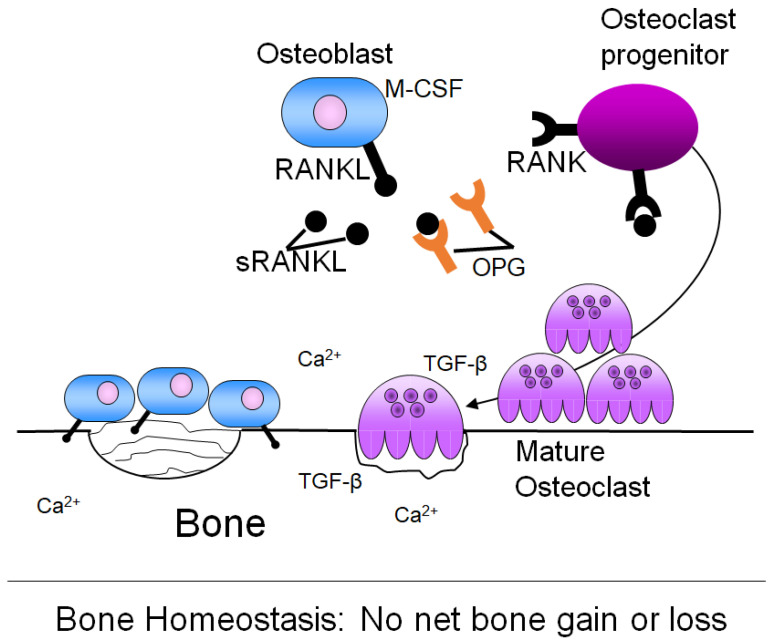
Bone homeostasis. In the healthy adult, there is a tightly regulated balance between osteoblast bone deposition and osteoclast bone resorption, such that there is no net bone gain or loss. To initiate bone resorption, osteoblasts express RANKL on their cell surface. Alternatively, osteoblasts can also express sRANKL. RANKL then binds the RANK receptor on osteoclast precursors, leading to formation of mature osteoclasts and bone resorption. M-CSF is another molecule produced by osteoblasts that can initiate osteoclast formation. OPG, also produced by osteoblasts, is a decoy receptor for RANKL that regulates osteoclast formation. Once bone resorption occurs, growth factors and minerals stored in the bone, including TGF-beta and/or calcium (Ca^2+^) are released. A signal is then sent to osteoblasts to migrate to the site of resorbed bone and synthesize bone matrix to fill in the hole. Thus, homeostasis is maintained, with no net bone gain or loss.

The relative activities of osteoblasts and osteoclasts are normally tightly coupled and regulated in order to maintain a balance between bone formation and degradation (Figure 2). Cells in the bone marrow, especially stromal and immune cells, produce cytokines and growth factors that influence the activities of osteoblasts and osteoclasts [84,85]. However, this balance between bone synthesis and resorption is upset in several pathological conditions, including bone metastatic breast cancer, resulting in osteoclast activity in excess of bone deposition by osteoblasts and subsequently net bone loss [85].

**Figure 3:**
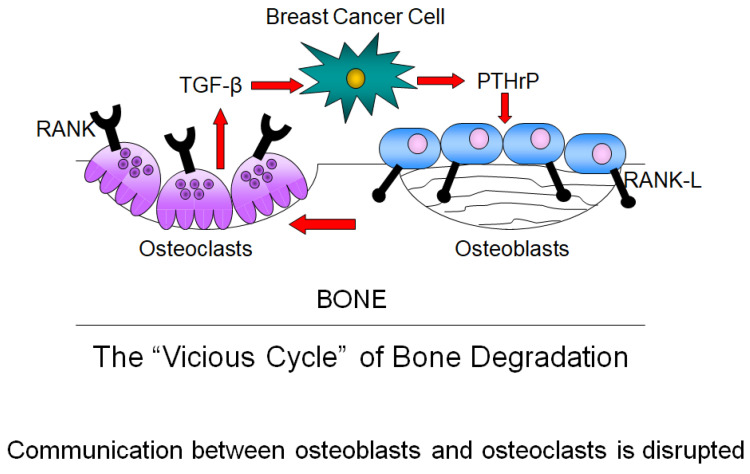
The “vicious cycle” of bone degradation. When breast cancer cells enter the bone microenvironment, they produce PTHrP that induces osteoblasts to increase their expression of RANKL. RANKL then binds the RANK receptor on osteoclast precursors, leading to formation of mature osteoclasts and bone resorption. Growth factors stored in the bone, including TGF-beta are released, which feed back onto breast cancer cells to continue their release of PTHrP. This feed-forward loop causes an overactivation of osteoclast formation and sustained bone resorption. Osteolytic lesions occur due to an inability of the osteoblasts to deposit bone matrix and fill the lesions.

Breast cancer metastasis to bone disrupts the tightly regulated balance between osteoblasts and osteoclasts. In the original model proposed by Guise, breast cancer cells overproduce PTHrP [86,87]. This then activates osteoblasts to produce RANKL, which binds to the RANK receptor on osteoclast precursors, inducing osteoclast formation and bone matrix destruction. TGF-β is released from the destroyed bone matrix and stimulates cancer cells to produce more PTHrP [82]. This feed-forward loop establishes a “vicious cycle”, resulting in constitutive osteoclast activation, an inability of osteoblasts to lay down bone matrix, and sustained bone degradation (Figure 3) [88,89]. Ultimately, osteolytic lesions form at sites of metastases [90,91], which lead to intractable bone pain and pathologic fractures [92]. Breast cancer can rarely also cause osteoblastic or mixed osteoblastic-osteolytic disease, but the great majority are osteolytic—about 85 percent of all breast cancer bone lesions [92].

### Adjuvant Bone-Modifying Agents for Early Hormone Receptor-Positive Breast Cancer

Bone-modifying agents are used to ameliorate the therapy-induced loss of BMD seen in patients treated with cytotoxic chemotherapy and endocrine therapy. In addition, in the adjuvant setting, bone-modifying agents administered at the time of primary treatment play a role as preventive measures against later bone recurrence and fractures [9]. The first class of antiresorptive drugs are bisphosphonates such as alendronate, risedronate, ibandronate, and zoledronic acid. Bisphosphonates are highly effective antiresorptive agents composed of synthetic pyrophosphate analogs that bind strongly to the bone mineral and incorporate into the bone matrix, where they can remain pharmacologically effective for many years [93]. When osteoclasts attempt to resorb bone mineral, they also endocytose the bisphosphonates, which contain a molecular bond that the osteoclast is unable to hydrolyze. Ultimately, this perturbs mature osteoclast intracellular metabolism and induces osteoclast apoptosis, preventing osteoclast-mediated bone resorption [93]. Incorporating bisphosphonate therapy during primary cancer treatment can help prevent bone loss and mitigate the increased bone remodeling associated with cytotoxic chemotherapy and endocrine therapy [94]. 

For breast cancer patients who are postmenopausal (natural or induced), treatment with adjuvant bisphosphonates reduces overall cancer recurrence, distant recurrence, bone recurrence, and, importantly, increases disease-free and overall survival by around 25–30% [95]. However, the same anti-cancer benefits are not observed in premenopausal patients who retain ovarian function and concomitant estrogen production [66]. The reason for this disparity remains unclear, but preclinical evidence in mouse models suggests that the presence of reproductive hormones can prevent bisphosphonate-mediated cancer inhibition. In mice with disseminated breast cancer cells, administration of zoledronic acid only reduced tumor growth in ovariectomized mice, which represent surgical menopause, and not in mice with intact ovaries [96]. This supports the evidence seen in large-scale human clinical trials, which demonstrated that treating premenopausal patients with bisphosphonates had no effect on overall cancer recurrence, distant recurrence, bone recurrence, disease-free survival, or overall survival. As such, adjuvant bisphosphates are only considered for patients who are postmenopausal at the time of treatment [9].

The other antiresorptive agent used in clinical practice is denosumab, a human monoclonal antibody that binds the cytokine RANKL, which is normally produced by osteoblasts and binds to osteoclasts to induce osteoclast maturation and proliferation. By binding to RANKL, denosumab prevents its interaction with osteoclast precursors, halting osteoclast maturation and proliferation. RANKL inhibition therefore reduces osteoclast-mediated bone resorption [97]. Despite early evidence that postmenopausal early breast cancer patients treated with AIs and adjuvant denosumab show a reduction in clinical fractures [98] and disease-free survival [99], a large randomized trial showed that denosumab did not improve disease-related outcomes above the known beneficial effects of denosumab on skeletal health [100]. Therefore, denosumab is not currently recommended in the adjuvant setting [9].

## 5. Progression to Metastasis

### 5.1. The Metastatic Cascade

Cancer cell dissemination to secondary organs occurs through a series of coordinated steps. First, the growing primary tumor undergoes diversification through phenotypic instability to generate variant cell(s) with metastatic properties [101,102]. Proliferation of the primary tumor may be supported by growth factors produced by the primary niche or produced by the tumor. Next, the formation of new blood vessels occurs, which provide an endless supply of nutrients to the tumor. Subsequently, local invasion occurs, whereby the extracellular matrix is destroyed via matrix metalloproteinases produced by the tumor cells [101]. Tumor cells additionally experience an increase in motility and reduction in adherence-dependence, which facilitates their invasion. It is postulated that one way this conversion may occur is through epithelial-to-mesenchymal transition [103]. Next, the invasive cancer cells gain access to the circulation, either by direct entry into a blood vessel or via indirect access through the lymphatic system [101]. The process by which cancer cells gain access to the circulation is called intravasation. Intravasation can occur as a result of chemotaxis in response to a soluble chemotactic factor gradient or by physical pressure [103]. 

Chemokines and cytokines have been heavily implicated in cancer cell chemotaxis to bone as a secondary site [104,105,106,107]. Elevated levels of IL-8 production by human breast cancer cells have been correlated with increased bone metastasis in vivo [105,107]. Furthermore, IL-8 has been implicated with enhanced cell motility, invasion, and metastatic potential [108,109,110]. As another example, increased Monocyte Chemoattractant Protein-1 (MCP-1) expression has been associated with increased cancer cell proliferation and invasion [111,112,113]. MCP-1 has also been implicated as a chemoattractant for metastatic cancer cells [114]. As a final example, InterLeukin-6 (IL-6) has been shown to increase the migration of breast cancer cells, and it also enhances the survival of invasive tumor cells by acting as an anti-apoptotic factor [115,116].

After circulating through the vasculature, and/or responding to a chemoattractant gradient, disseminated tumor cells adhere to the vascular endothelium in their secondary site. Extravasation into the secondary niche then occurs, facilitated by additional cancer cell production of matrix metalloproteinases that destroy the surrounding tissue [101]. For bone in particular, disseminated tumor cells enter the bone in blood vessels called vascular sinusoids. Blood flow within the sinusoids is sluggish, which allows for the normal movements of lymphoid and hematopoietic cells [46,117]. However, this sluggish blood flow also enables disseminated tumor cells to easily enter the bone niche [46]. Breast cancer cells frequently metastasize to long bones, including the femur, whereby vascular sinusoids are located near the bone ends, regions of high metabolic bone turnover [82,118]. Growth factors, cytokines, and chemokines, including TGF-beta, Insulin Growth Factor (IGF), Bone Morphogenetic Proteins (BMPs), IL-6, and InterLeukin-8 (IL-8), are found in abundance as part of normal bone remodeling, making this region of the bone an especially attractive site for metastatic cell survival [118]. Once in the secondary site, disseminated tumor cells must have the ability to respond to local growth factors in order to colonize, grow, and survive [46]. Responding to factors present in the secondary microenvironment is a major rate limiting step in the metastatic cascade and distinguishes a metastatic cancer cell from a cell solely capable of dissemination without colonization [46,119].

### 5.2. Clinical Metastatic Recurrence

Despite best therapeutic efforts, some patients with primary breast cancer will progress to metastatic disease. Unlike other subtypes of breast cancers in which risk of recurrence decreases 5 years after diagnosis, the recurrence risk for HR-positive breast cancer remains steady from 5 years to at least 20 years, with some patients experiencing recurrence three decades after initial diagnosis [7]. Therefore, long-term monitoring is a critical part of breast cancer clinical care. Regular history and physical examinations are recommended every 4 to 6 months for the first 5 years after primary therapy and annually thereafter, in conjunction with annual mammography [9]. However, without any clinical evidence of metastatic disease, evaluation of serum tumor markers, routine bone scans, Computed Tomography (CT) scans, Magnetic Resonance Imaging (MRI) scans, Positron Emisson Tomography (PET) scans, or ultrasound examinations in the asymptomatic patient provide no survival advantage and are, therefore, not recommended [9,120]. Instead, patients should be instructed to monitor for signs of recurrence [9]. 

**Figure 4:**
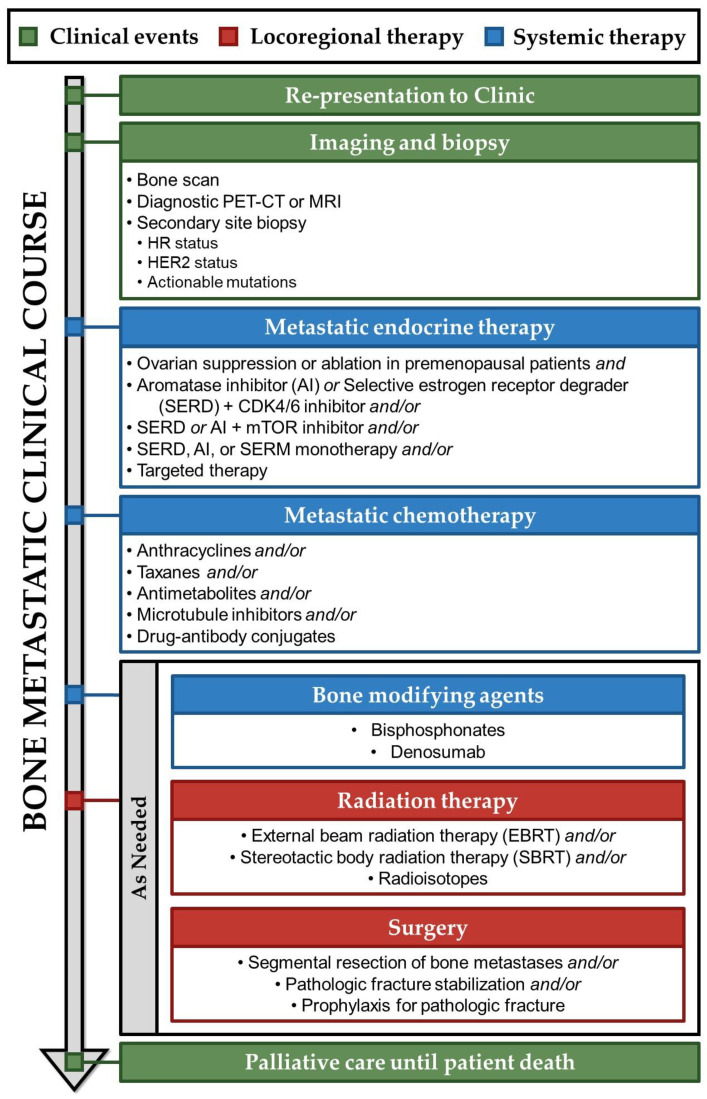
Representative timeline of bone metastatic breast cancer clinical course. A patient presents to the clinic with metastatic disease following signs or symptoms of recurrence or findings on history and physical examination. Diagnostic imaging and biopsy, preferably of the secondary site, are performed to discern molecular characteristics of the metastasis, which may differ from the original primary cancer. A trial of endocrine therapy is then performed in concert with administration of bone modifying agents. If progression occurs on endocrine therapy, chemotherapy is administered. Palliative radiation therapy or surgery are used to control pain or pathologic fracture within the metastatic site and may be administered at any time along the patient’s clinical course. Finally, when disease progresses or the burdens of treatment outweigh the benefits, palliative care is provided to the patient until death.

Among patients who develop metastatic breast cancer, 45% experience their initial metastasis in the bone, a considerably higher occurrence than in the lungs (19%), liver (5%), or brain (2%) [121]. Patients with bone metastatic breast cancer may present with bone pain, pathologic fractures, symptoms of spinal cord compression including incontinence and muscle weakness, or hypercalcemia, which can cause nausea and altered mental status [122]. Each of these signs in a patient with a history of breast cancer warrants further follow-up and begins the clinical course outlined in Figure 4.

Upon a patient presenting to the clinic, a history, physical exam, and blood tests should be obtained, including liver function tests and alkaline phosphatase levels. Alkaline phosphatase is a serum marker of both hepatobiliary pathology and bone turnover and mineralization [123]. Bone-specific alkaline phosphatase is expressed on the cell surface of osteoblasts, and serum levels correlate with increased osteoblastic activity, which occurs in both osteoblastic and osteolytic lesions [86,123]. In osteoblastic lesions, this elevation correlates with local stimulation of osteoblasts, while in osteolytic lesions, it is secondary to local bone destruction and subsequent compensatory bone formation [86]. Patients with active bone metastases typically have elevated levels of alkaline phosphatase and should be followed with skeletal scintigraphy (bone scan), radiographs of any long bones that are painful or appear abnormal on bone scan, and diagnostic PET-CT or MRI [9]. 

Biopsy tissue should be acquired, if possible, from the site of recurrence and tested for molecular characteristics, HR status, and staging, as in primary disease [9]. Importantly, metastatic tumors may differ from the primary lesion, especially in HR and HER2 expression. In a large-scale study, 16% of patients who previously had HR-positive, HER2-negative primary tumors had changes in HR/HER2 expression in metastatic sites [124]. Of this 16%, 67% lost HR receptors, 25% gained HER2 receptors, and 8% both lost HR and gained HER2 receptors [124]. HR discordance with loss of HR status was significantly associated with a worse overall survival and has implications for therapeutic options [124]. Therapy choices can also be guided by tumor biomarkers. Unlike in primary disease, where tumor genomics are indirectly assessed by multigene assay, metastatic breast cancer is tested directly for actionable somatic mutations, in order to determine a patient’s eligibility for targeted therapy. For example, demonstrable mutation in *PIK3CA* using Next-Generation Sequencing (NGS) of tumor tissue or Circulating Tumor Deoxyribonucleic Acid (ctDNA) in blood renders a patient eligible for second-line Phosphatidylinositol-4,5-Bisphosphate 3-Kinase (PIK3) inhibitors, including alpelisib [125]. *PIK3CA* or AKT Serine/Threonine Kinase 1 (*AKT1*) activating mutations or *PTEN* alterations detected by NGS determine eligibility for the AKT inhibitor capivasertib, and acquired Estrogen Receptor 1 (*ESR1*) mutations at recurrence or progression on endocrine therapy make a patient eligible for the estrogen receptor antagonist elacestrant [25]. Clinicians may also test for Neurotrophic Receptor Tyrosine Kinase (*NTRK*) fusions, microsatellite instability-high/deficient mismatch repair biomarkers, tumor mutational burden, and Ret Proto-Oncogene (*RET*)-fusion in certain circumstances [125].

## 6. Treatment of Bone Metastatic Breast Cancer

Metastatic breast cancer treatment shares many features with primary treatment. However, treatment plans are more highly individualized, focusing on a patient’s specific site of recurrence for symptom relief and slowing of disease progression. It is estimated that about half of patients with bone relapse present with bone-only metastases and half present with other organ involvement [17]. Compared to patients with visceral involvement, patients with bone-only metastases have markedly longer overall survival, and a minority never develop extra-osseous metastases along their disease course [126]. For some patients with oligometastatic, bone-only disease, aggressive locoregional therapy may be pursued with curative intent [127]. However, most patients with bone metastases are treated extensively with endocrine therapy, chemotherapy, bone-modifying agents, and locoregional therapy to reduce or prevent tumor growth in both skeletal and extra-skeletal sites [126,128]. 

In the widely metastatic stage, breast cancer is treatable, but is not generally considered curable [9]. Therefore, treatment objectives shift to optimizing patient quality of life and, if possible, prolonging time to progression of disease and death [129]. Treatments with minimal toxicity are consequently preferred to maintain patient quality of life, and, except in cases of immediately life-threatening organ dysfunction, a trial of endocrine therapy should be the initial treatment for patients with bone-metastatic HR-positive disease [130].

### 6.1. Endocrine Therapy for Bone Metastatic Breast Cancer

Metastatic endocrine therapy employs many of the same principles and therapies that are used in the adjuvant setting, with important additions. The objective of endocrine therapy remains to decrease circulating estrogen or ER activity and prevent estrogen-related tumor growth in hormone-responsive cancers [131]. Notably, treatment guidelines pertain to both postmenopausal patients and premenopausal patients, who require ovarian suppression or ablation. Premenopausal patients are frequently underrepresented in clinical trials for metastatic breast cancer, so treatment strategies for premenopausal patients are typically extrapolated from postmenopausal patient data, with the addition of ovarian suppression or ablation [132]. As previously discussed, both natural and induced menopause profoundly decrease BMD through rapid decline in estrogen levels [59]. 

HR-positive cancers commonly lose endocrine sensitivity over time [131]. As a result, the usual treatment approach involves a sequential strategy, where one endocrine therapy is pursued until intolerable toxicity or progression occurs, followed by a switch to another endocrine therapy [133]. The optimal sequence for endocrine treatment remains unclear and is influenced by factors such as previous treatments, tolerance, and patient preference [9]. First-line metastatic endocrine therapy commonly includes AIs, as in primary disease. AIs are typically administered with a Cyclin-Dependent Kinase (CDK) 4/6 inhibitor, such as palbociclib, ribociclib, or abemaciclib. Cyclin-dependent kinases 4 and 6 are enzymes that mediate transition through the cell cycle and guard genome integrity [134]. ER-positive breast cancer cells are reliant on CDK4/6 for cell cycle progression [135]. Inhibition of CDK4/6 in combination with AIs in metastatic HR-positive, HER2-negative breast cancer has been shown to improve progression-free survival and objective response rate compared to AI monotherapy [136,137,138]. However, recently reported trials suggest that overall survival benefits are not consistent among the three approved CDK4/6 inhibitors when combined with AIs [135]. Only the addition of ribociclib to AIs has yet been shown to increase overall survival over AIs alone [139]. Though no trials have yet reported the effects of CDK4/6 inhibitor on bone outcomes, preclinical data interestingly report that only ribociclib has no toxic effects on osteoblasts in-vitro. The preserved viability of osteoblasts in the bone–tumor microenvironment could play a role in the overall survival benefit associated with ribociclib plus AI [134].

As an alternative to AIs, CDK4/6 inhibitors may instead be combined with fulvestrant, a selective estrogen receptor degrader/downregulator (SERD) and ER antagonist [140]. This is particularly beneficial for patients who experience progressive disease during AI treatment or develop a recurrence within one year of adjuvant endocrine therapy [141]. Fulvestrant plus ribociclib [142] or abemaciclib [143] has been shown to increase overall survival over fulvestrant monotherapy. Fulvestrant may also be combined with an AI, which has been shown to improve overall survival over AI alone [144,145]. SERDs like fulvestrant induce accelerated degradation of ER, resulting in a pure antiestrogenic effect on HR-positive breast cancer cells [140]. While fulvestrant is a pure anti-estrogen, it does not appear to decrease bone mass, in contrast to other estrogen modulators such as SERMs and AIs. Though clinical data are surprisingly limited, fulvestrant administration does not seem to increase markers of bone turnover [146,147]. 

For patients with metastatic breast cancer, next-line endocrine therapy options are pursued when disease progression occurs on first-line therapeutic regimens. Often, this is due to acquired endocrine resistance, which is associated with aberrant activation of the Mammalian Target Of Rapamycin (mTOR) signal transduction pathway [148]. Co-targeting the mTOR pathway and ER can restore sensitivity to patients with endocrine therapy-resistant advanced breast cancer [149]. Everolimus is an oral inhibitor of mTOR that has been shown to increase progression-free survival when added to AI [148] or fulvestrant [150] and overall survival when added to tamoxifen [151]. It should be noted, however, that there have been some reports of therapeutic resistance to everolimus in patients with AI resistance and estrogen-depleted-resistant breast cancer [152]. Regardless, everolimus has been reported to have bone-protective effects in metastatic disease. In a large clinical trial, adding everolimus to the AI exemestane reduced bone turnover markers when compared to exemestane alone [153]. It also decreased progression of pre-existing bone lesions and development of new bone lesions [153]. This finding was supported by preclinical work that showed that everolimus reduced osteoclast progenitor maturation in-vitro and osteoclast quantity and concomitant osteoclast-mediated bone resorption in an ovariectomized mouse model of metastatic breast cancer [154]. Other subsequent-line endocrine therapies may include SERD, SERM, or AI monotherapy, but these remain less well defined [9]. Providers and patients should engage in shared decision-making when considering subsequent lines of therapy and evaluate potential clinical risks, benefits, and patient goals and preferences. 

### 6.2. Chemotherapy for Bone Metastatic Breast Cancer

When resistance to endocrine therapies becomes evident, the use of chemotherapies and targeted treatments is appropriate [141]. Chemotherapy is also indicated for patients who have symptomatic or rapidly progressive visceral metastasis [9]. Various chemotherapy protocols are considered suitable, including sequential monotherapy or select combinations in certain patients. Although combination chemotherapy may provide a longer time to progression, it is also associated with greater toxicity and generally provides only marginal survival benefit [155]. Therefore, sequential monotherapy is typically preferred, except in patients with rapid, symptomatic disease progression [9]. Agents are continued until progression or unacceptable toxicity occurs, decided in concert with the patient.

Many of the same chemotherapeutic agents are used in both metastatic and primary breast cancer treatment. These include the anthracycline doxorubicin and the taxane paclitaxel. Other treatments are used primarily in the metastatic setting, including anti-metabolites, microtubule inhibitors, and antibody-drug conjugates. 

Anti-metabolites, which are incorporated into cancer cell nucleic acids and interfere with their synthesis, include capecitabine and gemcitabine [51]. Capecitabine is an oral, well-tolerated agent that increases objective response rate compared to traditional regimens [156] and improves quality of life in patients with heavily pretreated metastatic breast cancer [157]. An anti-metabolite alternative is gemcitabine, which is also efficacious in pretreated metastatic breast cancer patients [158]. A conjugate drug of gemcitabine and the bisphosphonate ibandronate was shown to localize to the bone microenvironment in a mouse model of osteosarcoma, a primary bone cancer [159]. When administered with docetaxel, the combination reduced tumor burden and improved mouse survival, while preserving bone architecture as measured by microCT. This innovative approach allows for targeted drug delivery to the bone microenvironment, allowing lower and less toxic doses of systemic drugs to be administered.

Microtubule inhibitors, which disrupt cell division in cancer cells, include eribulin and vinorelbine [51]. Eribulin is indicated for patients who have previously received an anthracycline, a taxane, and at least two chemotherapeutic regimens for the treatment of metastatic disease [9]. Importantly, single-agent eribulin has been demonstrated to increase overall survival in patients with heavily pretreated metastatic breast cancer over physician’s choice of chemotherapy [160]. However, in a recent preclinical study, mice treated with eribulin experienced a decrease in BMD as a result of increased osteoclast numbers and activity. The mechanism for this is thought to be indirect, with eribulin treatment causing a decrease in the RANKL decoy receptor OPG. With diminished OPG, the relative availability of RANKL increases, allowing the RANK/RANKL system to promote increased osteoclastogenesis [161]. Unfortunately, no clinical data have yet been reported regarding eribulin effects on bone in humans, but this preclinical study suggested that eribulin may cause bone loss in patients with metastatic breast cancer. An additional microtubule inhibitor option is vinorelbine, which has also demonstrated survival benefits in previously treated metastatic breast cancer, but little data on its effects on bone have been reported [162]. 

Finally, recent evidence has supported the use of drug–antibody conjugates in metastatic breast cancer. Sacituzumab Povitecan (SG) delivers SN-38, a topoisomerase I inhibitor, linked to an antibody targeting Trophoblast cell-surface antigen 2 (Trop-2), an epithelial antigen highly expressed in breast cancer [163]. SG is newly indicated for patients with metastatic unresectable breast cancer who are refractory to endocrine therapy and have received at least two prior lines of chemotherapy for metastatic disease [164]. In this patient population, SG has shown improvement in progression-free survival over physician’s choice of chemotherapy, but overall survival results have not yet been determined [163]. Given its recent introduction into the clinic, no information has yet been reported regarding SG effects on bone.

### 6.3. Bone-Modifying Agents for Bone Metastatic Breast Cancer

Endocrine and chemotherapeutic agents have profound effects on the bone microenvironment, and most therapies in the metastatic setting ultimately reduce BMD. As in adjuvant treatment of primary breast cancer, bone-modifying agents can help ameliorate therapy-induced loss of BMD, and their administration is standard of care in treating patients with bone metastatic breast cancer with life expectancy beyond 3 months [9]. Bisphosphonate usage has been rigorously demonstrated to reduce the risk of Skeletal Related Events (SREs) and bone pain and increase the time to SRE development [165]. However, unlike in the adjuvant setting where bisphosphonate administration has been shown to increase overall survival, bisphosphonates in metastatic breast cancer are a palliative measure only: no survival benefits have been demonstrated [165]. Compared to bisphosphonates, denosumab further reduces the risk of SRE by 22%, but demonstrates no additional benefits for time to SRE, bone pain, or overall survival [165].

### 6.4. Radiation Therapy for Bone Metastatic Breast Cancer

Radiation therapy has historically been used in bone metastatic breast cancer for analgesic purposes. The exact mechanism of RT-induced pain relief remains unclear, but it is thought that decreased tumor burden alleviates the mass effect on surrounding nerve structures [166] and decreased osteoclast-mediated bone destruction relieves inflammatory pain [167,168]. Evidence suggests that, beyond pain palliation, RT may have a role in SRE prevention. In a recent study, patients with asymptomatic high-risk bone metastases of either lung, breast, prostate, or other cancers were treated with either prophylactic RT or standard of care without RT. Patients who received prophylactic RT had significantly fewer SREs and improved overall survival [169]. Though a larger clinical trial is warranted, these results may suggest a wider role for RT in bone metastatic breast cancer beyond palliation.

Patients with bone metastatic breast cancer receive RT in a number of ways, including External Beam Radiation Therapy (EBRT), Stereotactic Body Radiation Therapy (SBRT), and through radioisotopes. EBRT is the standard of care for patients with painful bone metastases [169]. Compared to EBRT, SBRT administers a higher dose fraction of radiation but is more highly localized to its target, sparing nearby structures. SBRT is a reasonable option for patients with limited metastatic disease, with the intention of eradicating all known active disease and preventing disease progression [170]. Additionally, SBRT is used in the treatment of spinal metastases, where precise localization is critical to avoid excess irradiation of the spinal cord [170]. Increasingly, SBRT is being adopted for treatment of non-spinal bone metastases, but data in this setting are limited and outside the scope of professional society guidelines [171,172]. Studies comparing EBRT and SBRT for control of bone pain have produced mixed results, with some finding SBRT superior and others showing no appreciable difference between the two methods [173]. 

For patients with diffuse bone pain who are not eligible for palliative local RT with EBRT or SBRT, radioisotopes may be indicated [174]. Radioisotopes are given systemically and are localized to sites of active bone turnover, emitting high-energy β particles to provide cytotoxic irradiation of nearby cancer cells [175]. In the United States, strontium-89 is FDA-approved for treatment of painful bone metastases and samarium-153 is approved exclusively for osteoblastic bone lesions, which are present in some metastatic breast cancer cases [175]. Radium-233 is approved for pain relief in castration-resistant prostate cancer, where it also has been demonstrated to increase overall survival [176]. However, it remains poorly evaluated in metastatic breast cancer. Isolated case reports suggest that it may provide effective and tolerable pain relief, though with no reported survival outcomes [176].

### 6.5. Surgery for Bone Metastatic Breast Cancer

Beyond RT, surgery can be effective in ameliorating pain and responding to SREs, though with considerably more morbidity. Because the bone microenvironment surrounding skeletal metastases undergoes aberrant remodeling, pathological fracture healing is greatly impaired compared to normal bone [177]. As a result, most pathological fractures require surgical fixation for stabilization [178]. Surgical intervention to address impending fractures provides improved outcomes [178], but providers should strongly consider the life expectancy of the patient before undertaking preventative surgery requiring extensive recovery [178].

## 7. Palliative Care until Patient Death

Together, a patient and provider decide whether disease is being controlled and the toxicity of treatment is acceptable [179]. Should the burden of anticancer therapy outweigh its benefits, goals of care, prognosis, and end-of-life care should be discussed. Ideally, a multidisciplinary team consults to address physical, psychosocial, and practical interventions. The goal for each patient is to provide a death free from avoidable distress and suffering for patients, families, and caregivers; in general accord with the patient’s and family’s wishes; and consistent with clinical, cultural, and ethical standards [179]. The 5-year survival rate for patients with metastatic breast cancer is 29% [1].

## 8. Concluding Remarks

Patients diagnosed with HR-positive early breast cancer undergo aggressive treatment in hopes of eradicating local disease and preventing future recurrence. While treatment for early breast cancer is generally very effective, most of the therapeutic agents have deleterious effects on skeletal health that lead to bone remodeling and lowering of BMD. These skeletal changes wrought by chemotherapy and endocrine therapy may predispose patients to future bone metastases. In the adjuvant setting, antiresorptive bisphosphonates have been demonstrated to reduce this risk in postmenopausal women.

Despite advances in early breast cancer treatment, some patients progress to metastatic disease, and the most common site for dissemination is the bone. HR-positive/HER2-negative tumors are particularly associated with bone relapse. Patients with HR-positive bone metastatic breast cancer encounter distinct challenges to the integrity of their skeletal system. Initially, the widespread presence of cancer in the bone triggers a destructive local cycle. Subsequently, patients undergo endocrine therapy that requires elimination of estrogen signaling, abolishing estrogen’s protective effects on bone. Lastly, chemotherapy contributes to a further reduction in BMD, collectively rendering patients susceptible to SREs including pathologic fractures, intractable pain, and spinal cord compression. 

The treatment approach for patients with bone metastatic breast cancer requires a highly individualized approach. Patients may be eligible for multiple lines of therapy, and clinicians therefore have discretion in choosing an appropriate treatment strategy. Therapy choice is dependent on a given patient’s previous treatments and response. For example, one patient may be successful with an AI and CDK4/6 inhibitor in the first-line metastatic setting, while another patient who previously had disease progression on adjuvant AI may be instead offered fulvestrant with a CDK4/6 inhibitor as a first-line metastatic treatment. Practitioners also must consider if an individual patient is eligible for targeted therapies based on tumor-specific somatic mutations. For example, PIK3 inhibitors like alpelisib or the novel AKT inhibitor capivasertib have been demonstrated to increase survival only in patients with *PIK3CA*, *AKT1*, or *PTEN* mutations. Identifying which patients may benefit from which therapies is a critical component of metastatic breast cancer care. Additionally, bone metastatic breast cancer can cause a variety of clinical sequelae, which may present differently in each patient. For instance, while some patients may experience spinal instability, others may develop hypercalcemia, and care must address each patient’s individual needs in symptom management and palliation. Finally, each patient has unique goals, values, and preferences for metastatic breast cancer treatment. Some patients may wish to continue anticancer treatment for as long as possible, and others elect to focus care on maintaining quality of life. Practitioners and patients must engage in shared decision-making to collectively decide on the best course of individual treatment.

Clinicians have a diverse armamentarium of therapies at their disposal to combat metastatic breast cancer and palliate symptoms, including endocrine therapy, chemotherapy, bone-modifying agents, and radiation therapy. Despite this, bone metastatic breast cancer remains an incurable disease, though several effective treatment strategies have emerged that extend progression-free survival and overall survival. CDK4/6 inhibitors have been heralded as a breakthrough since their introduction into the clinic and have provided the single greatest improvement in overall survival of any therapy for advanced HR-positive, HER2-negative breast cancer to date. The addition of CDK4/6 inhibitor ribociclib to AI therapy was demonstrated to improve overall survival for patients with metastatic breast cancer by more than 12 months over AI alone [139]. CDK4/6 inhibitors have proven so effective in metastatic settings that abemaciclib has been newly approved for HR-positive/HER2-negative early-breast cancer patients at high-risk of recurrence [180,181]. In this way, CDK4/6 inhibitors represent an ongoing strategy in combatting metastatic breast cancer; optimizing primary breast cancer treatment in order to prevent metastasis development.

Despite their efficacy, most patients eventually develop resistance to CDK4/6 inhibitors. To address this, a current major endeavor in metastatic breast cancer research focuses on overcoming or preventing therapy resistance. A novel small molecule PI3K inhibitor, inavolisib, has more than doubled progression-free survival when combined with CDK4/6 inhibitor palbociclib plus fulvestrant in patients with recurrent *PIK3CA*-mutated, HR–positive, HER2-negative advanced breast cancer [182]. Though no results are yet available regarding overall survival outcomes, this combinatorial strategy incorporating targeted therapy for eligible patients is among the most promising treatment options. Likewise, targeted therapy such as AKT inhibitor capivasertib in patients with *PIK3CA*, *AKT1*, or *PTEN* mutations, or estrogen receptor antagonist elacestrant in patients with acquired *ESR1* mutations have improved progression-free survival in those patient populations [183,184]. Precision therapy based on individual tumor characteristics is a growing part of next-generation metastatic cancer care. 

Unlike targeted therapy, immunotherapy has had little success in treatment of HR-positive, HER2-negative breast cancer, despite revolutionizing the treatment of other cancer types. Immune checkpoint inhibitors such as pembrolizumab, which have been successful in other cancers, are currently only indicated for metastatic breast cancer patients with MicroSatellite Instability-High (MSI-H) or MisMatch Repair deficient (dMMR) tumors, having shown little efficacy in other HR-positive populations [9]. However, another type of immunotherapy, antibody-drug conjugates, have shown promise using antibodies to specifically target breast cancer cells and deliver chemotherapy. Sacituzumab govitecan is the first of its class in HER2-negative breast cancer and represents a next-generation approach to cancer care. 

As advances in the treatment of metastatic breast cancer lead to extended patient survival, care and consideration need to be given to preserving skeletal health and enhancing the overall quality of life for patients as they navigate an extended lifespan.

## Data Availability

Not applicable.
